# Malignant transformation of mature ovarian teratoma into goblet cell adenocarcinoma: a case report

**DOI:** 10.1016/j.gore.2026.102167

**Published:** 2026-07-10

**Authors:** Chinmayi Aryasomayajula, Nichole Legaspi, Mai Elzieny, Hanlin L. Wang, Ritu Salani, Peggy S. Sullivan

**Affiliations:** a University of California, Los Angeles, Division of Gynecology Oncology, United States of America; b University of California, David Geffen School of Medicine, United States of America; c University of California, Los Angeles, Division of Pathology and Laboratory Medicine, United States of America

**Keywords:** Mature cystic teratoma, Malignant transformation, Goblet cell adenocarcinoma, Immunohistochemistry, Case report

## Abstract

**Background:**

Malignant transformation of ovarian mature cystic teratomas (MCTs) occurs in 0.17–2% of cases, with squamous cell carcinoma comprising over 80%. Transformation to goblet cell adenocarcinoma is exceedingly rare, and optimal management remains undefined.

**Case:**

A 39-year-old woman with a history of prior ovarian cystectomy for MCT presented with severe abdominal pain and bilateral adnexal masses. Tumor markers were elevated (CA-125 76 U/mL, CEA 175 ng/mL, CA 19–9139 U/mL). She underwent total abdominal hysterectomy, bilateral salpingo-oophorectomy, omentectomy, appendectomy, and surgical staging. Intraoperative findings included a 21 cm right and 15 cm left ovarian mass with hemoperitoneum. Final pathology demonstrated goblet cell adenocarcinoma arising from MCT with contralateral ovarian involvement. No tumor was identified in the appendix. Immunohistochemistry was positive for SATB2, CK20, and CDX2, and negative for CK7 and PAX8, consistent with intestinal-type differentiation, with focal synaptophysin positivity indicating neuroendocrine features. Postoperative imaging revealed suspicious retroperitoneal lymphadenopathy. Given that standard germ cell regimens target totipotent biology and platinum-taxane regimens address Mullerian-derived epithelial ovarian carcinoma — neither of which reflects the histology seen — a histology-directed approach favoring GI-based chemotherapy was adopted, treating by cell type rather than site of origin. She is currently on third line treatment given disease progression.

**Conclusion:**

Goblet cell adenocarcinoma arising in ovarian MCT is a rare entity without standardized treatment guidelines. The intestinal-type immunophenotype supports extrapolation from appendiceal goblet cell adenocarcinoma treatment paradigms, favoring gastrointestinal over traditional gynecologic chemotherapy regimens. Further collaborative reporting is needed to define optimal management for this rare tumor.

## Highlights

• Goblet cell adenocarcinoma within ovarian mature cystic teratoma is rare.
• Goblet cell-like and neuroendocrine features supports goblet cell adenocarcinoma.
• Mature cystic teratoma and clinical work-up can help establish an ovarian primary.
• Gastrointestinal-type chemotherapy may be favored over gynecologic regimens.

## 1 Introduction

Mature cystic teratomas (MCTs) are common ovarian neoplasms, accounting for approximately 20% of all ovarian tumors. Malignant transformation occurs in approximately 0.17–2% of ovarian MCTs, with squamous cell carcinoma representing over 80% of these cases (Hackethal et al., 2008; Neville et al., 2025). Malignant transformation most commonly occurs in postmenopausal women, and many teratomas are thought to be present for 15–20 years prior to undergoing secondary malignant transformation (Hackethal et al., 2008). However, cases have been reported across a wide age range, from adolescence to the eighth decade of life (Neville et al., 2025). The pathogenesis of malignant transformation in MCT remains incompletely understood. Because these tumors contain mature tissue derived from all three germ cell layers (ectoderm, mesoderm, and endoderm), malignant transformation can theoretically arise from any of these components (Hackethal et al., 2008). Transformation to adenocarcinoma, however, is exceedingly rare. Here, we present a case of mature cystic teratoma with malignant transformation to goblet cell adenocarcinoma. Informed consent was obtained from the patient for the publication of this case report and any accompanying images.

## 2 Case report

### 2.1 Initial presentation

A 39-year-old woman initially presented to an emergency department (ED) with severe abdominal pain. Her medical history was notable for a right ovarian cystectomy in 2014, with final pathology confirming a mature cystic teratoma, as well as uterine leiomyoma and a family history of endometrial cancer. Initial imaging with CT abdomen/pelvis showed a myomatous uterus and bilateral adnexal masses suspicious for mucinous borderline tumor, but no enlarged lymph noted were noted. Her pain was treated, and she was referred to our institution to establish follow up with gynecology. While awaiting outpatient follow up, she re-presented to the ED one month later with chest pain and was diagnosed with bilateral pulmonary emboli on CT Chest Angiogram and started on apixaban. On discharge, she was seen by benign gynecology and was promptly referred to gynecologic oncology for further evaluation. On examination, a large abdominopelvic mass was palpable to just below the costal margin. A pelvic MRI was done initially to characterize the known fibroids, but noted a large abdominal mass of adnexal origin, suspicious for mucinous borderline tumor or adenocarcinoma, with no comment on lymph nodes. Serum tumor markers were notable for CA-125 of 76 U/mL, CEA of 175 ng/mL, and CA 19–9 of 139 U/mL (Normal ranges: CA-125 0–35 U/mL, CEA <3.1 ng/mL, CA 19–9 0–35 U/mL). Given concern for malignancy, she was counseled and consented for surgical resection of masses, intraoperative frozen section, and possible full surgical staging for ovarian cancer.

### 2.2 Surgical procedure

Intra-operative findings included an examination under anesthesia confirming a large pelvic mass extending to just below the rib cage. Upon entry into the peritoneal cavity, approximately 1000 mL of hemoperitoneum was encountered.

The right ovary measured 21 cm and was adherent to the mesentery and anterior abdominal wall. The left ovary was enlarged to 15 cm. There were no gross peritoneal implants or visible upper abdominal disease. The omentum appeared matted, and the appendiceal tip was firm. The liver, spleen, bowel, pancreas, and peritoneal surfaces were otherwise grossly normal. The right salpingo-oophorectomy specimen was sent for a frozen section, which demonstrated invasive mucinous adenocarcinoma with signet ring features, favoring metastasis. Given this, a total abdominal hysterectomy with left salpingo-oophorectomy was subsequently completed. Other abnormal sites of disease were also resected including several enlarged reactive left pelvic lymph nodes were noted in the external iliac lymph node region, omentum, and the appendix. As the intra-operative suspicion was for a gastrointestinal primary with ovarian metastasis, a full ovarian cancer staging procedure was not completed.

The patient required a transfusion of three units of packed red blood cells intraoperatively and two additional units postoperatively in the post-anesthesia care unit, with stable hemoglobin levels thereafter. Her postoperative course was complicated by ileus, which was managed conservatively. By postoperative day six, she was meeting all postoperative milestones and was discharged home in stable condition.

### 2.3 Surgical pathology

**Fig. 1:**
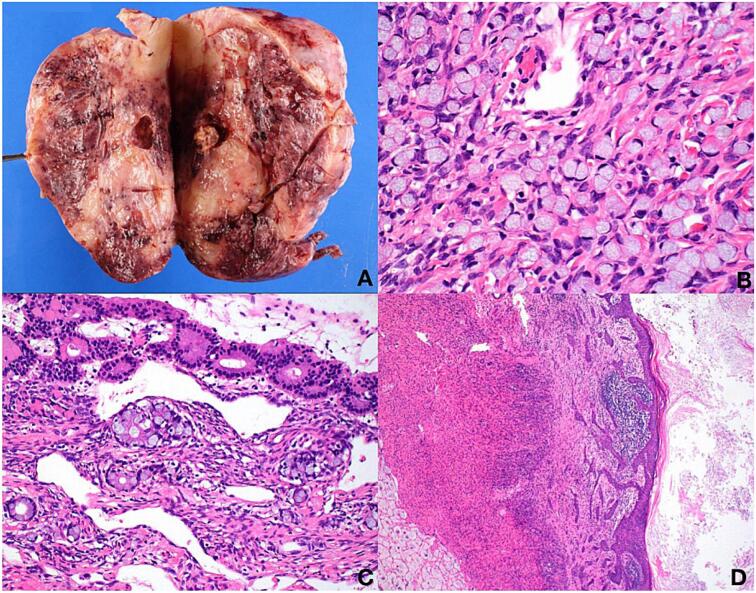
Gross and microscopic findings. A) Gross sections of the right ovarian mass show mostly solid red-tan tissue with extensive hemorrhagic and necrotic cut surfaces with focal cystic areas (gross photograph); B) Microscopic sections from the ovarian mass show an adenocarcinoma with areas of the tumor comprised of single signet-ring cells infiltrating the stroma (H&E stain, 400×); C) A microscopic section from the left ovarian cystic lesion shows a mature teratoma with intestinal differentiation and small clusters of goblet-like mucinous tumor cells associated with the intestinal crypts (H&E stain, 200×); D) A low-power microscopic image from the left ovarian mass shows mature squamous epithelium with keratinizing debris, characteristic of a mature cystic teratoma (right side of image), and adjacent extravasated mucin with floating tumor cells (lower left corner, H&E stain, 40×).

Sections of the right ovary showed mostly solid red-tan tissue with extensive hemorrhagic and necrotic cut surfaces (Fig. 1A). The left ovary had a nodular surface with mostly solid tan-white cut surfaces (not pictured). More extensive sampling of the adnexal masses was performed for permanent sections which included sampling of focal cystic lesions that were present bilaterally (Fig. 1A).

Microscopically, histologic sections from both adnexa showed parenchymal infiltration by tubules, clusters and cribriform groups and sheets of goblet-like mucinous cells. Single signet-ring cell infiltration was also seen (Fig. 1B). Extracellular mucin was abundant with tumor cell clusters floating within.

Sections of the cystic component of the left ovary showed a mature cystic teratoma with intestinal differentiation and small clusters of goblet-like mucinous tumor cells associated with intestinal crypts (Fig. 1C). The right ovary had extensive lymphovascular invasion and necrosis with focal features of a mature cystic teratoma. The nuclear atypia was mild-moderate with grade 2 tumor on the right adnexa (based on appendiceal goblet cell carcinoma grading). The mucinous tumor cells were immunohistochemically positive for SATB2, CK20, CDX2, and negative for CK7 and PAX8. Focal synaptophysin and INSM1 positivity was seen throughout the tumor. The appendix and other specimens were negative for malignancy. Findings were most consistent with goblet cell adenocarcinoma arising in an ovarian teratoma with metastasis to the opposite ovary (Fig. 1D).

### 2.4 Post-surgical course

She had a post-operative visit 20 days after surgery, at which time a referral to medical oncology was placed for discussion of adjuvant therapy for goblet cell adenocarcinoma arising in an ovarian teratoma with contralateral ovarian involvement. At that time, postoperative tumor markers demonstrated CA-125 of 71 U/mL, CEA of 11.7 ng/mL, and CA 19–9 of 31 U/mL. Circulating tumor DNA (ctDNA) testing was obtained by provider preference and based on colorectal data and was positive. Given the immunophenotypic overlap between mucinous ovarian tumors and gastrointestinal primaries, a thorough clinical workup—including imaging and endoscopic evaluation of the gastrointestinal tract—was performed to exclude an extra-ovarian primary mucinous malignancy; no alternative primary site was identified.

**Fig. 2:**
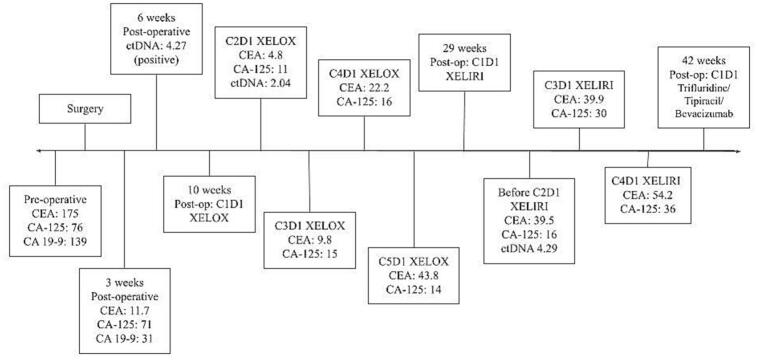
Trends in tumor markers throughout treatment. Clinical timeline illustrating the sequential treatment regimens and corresponding trends in serum tumor markers throughout the disease course. Reference ranges: CA-125, 0–35 U/mL; CEA, <3.1 ng/mL; CA 19-9, 0–35 U/mL.

Due to patient decision, she had her initial consult with a GI medical oncologist at our institution six weeks after surgery. At that time (∼7 weeks postop), a CT was done, revealing multiple prominent retroperitoneal and iliac chain lymph nodes suspicious for metastatic disease and an indeterminate 4 mm left upper lobe pulmonary nodule. She was then initiated systemic chemotherapy with medical oncology using a GI regimen given tumor histology approximately 10 weeks after surgery. She received five cycles of a XELOX regimen, which consists of oral capecitabine (1000 mg/m2 twice daily on Days 1–14) and oxaliplatin (IV 130 mg/m2 on Day 1) every 21 days. After five cycles of XELOX, interval imaging demonstrated disease progression, with enlargement of retroperitoneal lymph nodes, prompting a change in therapy from oxaliplatin- to irinotecan-based treatment. Her tumor markers were also noted to initially decline postoperatively but followed by a subsequent rise (Fig. 2). She was transitioned to a XELIRI regimen, consisting of oral capecitabine (1000 mg/m2 twice daily on Days 1–14), and irinotecan (IV 250 mg/m2 on Day 1) every 21 days. Serial ctDNA levels initially decreased from 4.27 to 2.04 over a two-month period during cycle 1–2 of XELOX; however, subsequent testing demonstrated an increase to 4.29 after 5 cycles of XELOX and 1 cycle of XELIRI. She received 4 cycles of XELIRI, and she was noted to be more symptomatic with abdominal pain and ascites, with interval imaging demonstrating progressive disease with enlarging retroperitoneal lymph nodes, right upper quadrant soft tissue thickening and moderate ascites. Systemic treatment was then changed to oral trifluridine/tipiracil (35 mg/m2 twice daily on Days 1–5 and 8–12) and bevacizumab (IV 5 mg/kg on Day 1 and 15). She has received one cycle of this regimen and given progression on two GI systemic chemotherapy regimens, she also is being evaluated for clinical trial consideration.

## 3 Discussion

Goblet cell adenocarcinoma arising in a mature cystic teratoma (MCT) of the ovary represents a rare form of malignant transformation and poses important diagnostic and therapeutic challenges. An exceedingly low number of cases have been reported in the literature including Baker *et al* who reported a series of “ovarian mucinous carcinoids” with 6 cases associated with MCT, single cases within larger cohorts, and isolated reports (Neville et al., 2025; Baker et al., 2001; Clark and Will, 2016; Huang et al., 2012). Consequently, the clinical behavior and optimal management of this entity remain poorly defined. This case contributes to the limited body of evidence and highlights key clinical and pathologic considerations.

Histologically, goblet cell adenocarcinoma demonstrates gastrointestinal-type mucinous differentiation characterized by goblet cell–rich epithelium. Goblet cell tumors may also exhibit neuroendocrine differentiation, and markers such as chromogranin, synaptophysin, and neuron-specific enolase may be expressed (Lancaster et al., 1997). Molecular studies have identified KRAS mutations in intestinal-type adenocarcinomas arising from MCT, suggesting that early carcinogenic events may parallel those seen in somatic gastrointestinal tissues (Hershkovitz et al., 2013).

The distinction between a primary or metastatic mucinous carcinoma involving the ovary can be difficult. While laterality, size, and immunohistochemistry may aid in the distinction, none of these features are diagnostic (Clark and Will, 2016; Yemelyanova et al., 2008). In our case, both markedly enlarged adnexa demonstrated parenchymal infiltration by signet-ring cells, goblet-like mucinous cells, and abundant extracellular mucin with focal neuroendocrine differentiation. The immunophenotype was consistent with intestinal-type differentiation, demonstrating CK20, SATB2, and CDX2 positivity with negative CK7 and PAX8 expression. This pattern contrasts with primary mucinous ovarian tumors of surface epithelial–stromal origin, which more commonly demonstrate strong CK7 expression with variable PAX8 and CK20 positivity (Clark and Will, 2016; Meagher et al., 2019; Tabrizi et al., 2010). With extensive and meticulous sampling of the gross specimen, we identified a focal MCT component with intestinal differentiation and goblet-like mucinous tumor cells associated with intestinal crypts, supporting an ovarian primary.

Due to the rarity of these tumors, standardized treatment guidelines have not been established. Management strategies are generally extrapolated from other malignant transformations of MCT as well as from appendiceal goblet cell adenocarcinomas. Surgical management typically includes comprehensive staging and cytoreductive surgery when feasible (Li et al., 2022). The role of adjuvant chemotherapy remains uncertain. For malignant transformation of MCT, platinum-based chemotherapy with paclitaxel and carboplatin has been the most commonly utilized regimen, with some studies demonstrating improved outcomes in patients with stage IC disease or higher (Qin et al., 2021). However, outcomes remain variable, with one case series reporting complete responses in 12 of 14 patients but a five-year overall survival of only 31.2% (Qin et al., 2021). In contrast, treatment paradigms for appendiceal goblet cell adenocarcinoma more commonly utilize gastrointestinal chemotherapy regimens. Current recommendations include capecitabine and oxaliplatin (XELOX) or folinic acid, fluorouracil, and oxaliplatin (FOLFOX) as first-line systemic therapy (Palmer et al., 2022). Given the intestinal-type differentiation and immunophenotype of goblet cell adenocarcinoma arising in MCT, extrapolation from appendiceal goblet cell tumors may be reasonable, with consideration of gastrointestinal chemotherapy regimens such as XELOX or FOLFOX. Although these doublet regimens are commonly extrapolated from colorectal cancer paradigms for the treatment of appendiceal goblet cell adenocarcinoma, it is important to note that these tumors are also rare and the limited available data suggest modest response rates of approximately 20–40% in appendiceal adenocarcinomas broadly, with GCA-specific partial response rates as low as 22% and disease progression observed in over half of treated patients (Palmer et al., 2022; Talia et al., 2022).

The selection of an appropriate chemotherapeutic regimen for intestinal-type adenocarcinoma arising in ovarian mature cystic teratoma (MCT) poses a significant therapeutic challenge. Standard germ cell regimens (*e.g.*, BEP) target totipotent germ cell biology and are inapplicable to terminally differentiated somatic malignancies. Likewise, platinum-taxane regimens used for epithelial ovarian carcinoma address Mullerian-derived neoplasms and do not reflect the histogenesis of intestinal-type differentiation within a teratoma. Accordingly, a histology-directed approach — treating by cell type rather than site of origin — was adopted, favoring fluoropyrimidine-based gastrointestinal regimens over traditional gynecologic protocols. This rationale is supported by the reclassification of teratoma-associated intestinal mucinous neoplasms under appendiceal and colorectal tumor taxonomy and by the identification of molecular alterations characteristic of colorectal carcinogenesis in these tumors (Talia et al., 2022).

Prognosis in malignant transformation of MCT is largely influenced by the FIGO stage at diagnosis and the ability to achieve complete cytoreduction. Patients with stage I disease generally have more favorable outcomes, whereas those with stage IC disease or higher demonstrate substantially worse survival (Neville et al., 2025). Most reported cases of intestinal-type adenocarcinoma arising within ovarian mature cystic teratomas have been confined to early-stage disease and treated with surgical resection alone. As a result, evidence to guide optimal systemic therapy for advanced or recurrent disease remains limited (Talia et al., 2022). Reported five-year overall survival in patients treated with adjuvant chemotherapy ranges from approximately 31% to 82% (Li et al., 2022; Qin et al., 2021). Tumor size may also influence recurrence risk, particularly when the malignant component exceeds 4 cm (Neville et al., 2025). Additionally, extrapolating from appendiceal goblet cell adenocarcinoma, high-grade histology and the presence of signet-ring cells are associated with poorer outcomes (Palmer et al., 2022; Yozu et al., 2018). Given that recurrences have been reported even in early-stage disease, long-term surveillance is warranted (Yozu et al., 2018).

Despite the biological rationale, our patient demonstrated a suboptimal response to two sequential GI-directed regimens, underscoring the limited evidence guiding treatment of this exceedingly rare entity and the need for further investigation into alternative therapeutic strategies, including molecular profiling–guided targeted therapy and immunotherapy. In cases such as these, multidisciplinary collaboration with gastrointestinal medical oncologists is essential, as their expertise in managing intestinal-type histologies can guide chemotherapy selection, treatment sequencing, and the integration of molecular profiling. Additional cases reported through collaborative studies and case reports will be critical to improving understanding of optimal management strategies and prognostic factors for this rare tumor.

## CRediT authorship contribution statement

**Chinmayi Aryasomayajula:** Writing – review & editing, Writing – original draft, Investigation, Conceptualization. **Nichole Legaspi:** Visualization, Investigation, Data curation. **Mai Elzieny:** Writing – review & editing, Visualization, Investigation, Data curation. **Hanlin L. Wang:** Writing – review & editing, Visualization, Investigation, Data curation. **Ritu Salani:** Writing – review & editing, Supervision, Investigation, Data curation, Conceptualization. **Peggy S. Sullivan:** Writing – review & editing, Supervision, Investigation, Formal analysis, Data curation, Conceptualization.

## Informed consent

Written informed consent was obtained from the patient for publication of this case report and accompanying images.
